# Real-World HbA1c Changes Among Type 2 Diabetes Mellitus Patients Initiating Treatment With a 1.0 Mg Weekly Dose of Semaglutide for Diabetes

**DOI:** 10.36469/001c.124111

**Published:** 2024-11-04

**Authors:** Noelle N. Gronroos, Caroline Swift, Monica S. Frazer, Andrew Sargent, Michael Leszko, Erin Buysman, Sara Alvarez, Tyler J. Dunn, Josh Noone

**Affiliations:** 1 Optum, Eden Prairie, Minnesota, USA; 2 Novo Nordisk Inc., Plainsboro, New Jersey, USA; 3 Novo Nordisk Inc, Plainsboro, New Jersey, USA; 4 New Jersey Psychological Association

**Keywords:** type 2 diabetes, GLP-1, real-world evidence, glycemic control, semaglutide

## Abstract

**Background:**

Medical management of patients with type 2 diabetes mellitus (T2DM) is complex because of the chronic nature of the disease and its associated comorbidities. Injectable once-weekly semaglutide for diabetes (OW sema T2D) is a type of glucagon-like peptide–1 receptor agonist approved for the treatment of patients with T2DM.

**Objectives:**

To describe patient characteristics and HbA1c changes for patients prescribed 1.0 mg maintenance dose OW sema T2D.

**Methods:**

This retrospective study included adult patients with T2DM with a pre-index glycated hemoglobin (HbA1c) of at least 7%, initiating treatment with OW sema T2D between January 1, 2018, and December 31, 2019, and prescribed a 1.0 mg maintenance dose. Patients were identified in the Optum Research Database and were included if they had continuous health plan enrollment for at least 12 months prior to (pre-index) and at least 12 months following (post-index) the date of the first OW sema T2D claim (index). Dose at initiation and prescriber specialty were captured. Change in HbA1c between the latest post-index and pre-index HbA1c measurement was calculated among all patients and among those with at least 90 days of continuous treatment (persistent patients).

**Results:**

A total of 2168 patients were included in this study. On average, patients were taking 13.5 different classes of medications. The majority of patients had lipid metabolism disorder (90.8%), hypertension (86.6%), diabetes with complications (86.8%), or other nutritional/endocrine/metabolic disorders (72.5%). The mean HbA1c reduction was 1.2% (P < .001). Patients persistent with OW sema T2D (n =1280) had a mean HbA1c reduction of 1.4% (P < .001). The mean (SD) days covered with a 1.0 mg maintenance dose was 236.1 (94.1) days.

**Discussion:**

Despite being medically complex, the patients in this real-world study experienced significant reductions in HbA1c following initiation of OW sema T2D.

**Conclusions:**

A 1.0 mg maintenance dose of OW sema T2D is an effective treatment for T2DM in the real world.

## BACKGROUND

Type 2 diabetes mellitus (T2DM) is a chronic metabolic disorder characterized by insulin resistance that has resulted in challenges to healthcare systems globally due to its increasing prevalence.^1,2^ In 2021, an estimated 29.7 million adults in the United States (US) were diagnosed with diabetes, with an additional 8.7 to 9.8 million adults estimated to remain undiagnosed.^3^ This high prevalence has contributed to extensive morbidity and mortality, making this chronic condition the eighth leading cause of death in 2022.^3^ Additionally, the economic burden is significant, with diabetes-related costs estimated at $412.9 billion in 2022, mainly due to complications such as cardiovascular disease, neuropathy, retinopathy, and nephropathy.^4^

The American Diabetes Association (ADA) has identified glycated hemoglobin A1c (HbA1c) as the key biomarker for measuring long-term glycemic control in patients with diabetes.^5,6^ Achieving an HbA1c target of less than 7.0% has been associated with a significant reduction in the risk of diabetes-related complications.^5^ However, the management of T2DM is still described as complex, particularly due to its multifactorial causes, chronic nature, and the frequent presence of comorbid conditions.^7,8^ Therefore, guidelines from the ADA recommend person-centered and integrated long-term treatment approaches.^7^ When selecting medications for patients with T2DM, guidelines recommend a patient-centric approach that includes the consideration of factors such as comorbidities, hypoglycemia risk, effect on body weight, side effects, cost, and patient preferences.^9,10^

Injectable once-weekly semaglutide for diabetes (OW sema T2D) is a glucagon-like peptide-1 receptor agonist (GLP-1 RA) that has demonstrated considerable efficacy in improving glycemic control and promoting weight loss in patients with T2DM. Administered subcutaneously once weekly, OW sema T2D has been shown to significantly reduce HbA1c levels and body weight.^11–16^ Clinical trials and meta-analyses have consistently reported that semaglutide provides substantial improvements in glycemic control and weight reduction compared with placebo and other antidiabetic agents.^11–19^ For example, a meta-analysis of the SUSTAIN trials showed that a 1.0 mg weekly dose of OW sema T2D reduced HbA1c by 1.5% to 1.8% after 30 to 56 weeks.^20^ Additionally, OW sema T2D had a positive impact on body weight, showing greater reductions in body weight than different comparators such as dipeptidyl peptidase 4 (DPP-4) inhibitors, sodium-glucose cotransporter-2 (SGLT-2) inhibitors, daily subcutaneous GLP-1 RAs, other once-weekly subcutaneous GLP-1 RAs, and insulin medications tested in the trials.^19,20^ Similarly, a meta-analysis by Zhong et al of phase 3 randomized controlled trials of OW sema T2D found that a 1.0 mg dose decreased HbA1c by 1.37%.^17^

Despite these results in clinical trial settings, real-world evidence remains limited. Real-world studies have become relevant in the decision-making process as they can reinforce clinical trial findings and provide insights into the medication’s performance in routine clinical practice.^21,22^ Although previous real-world studies have described HbA1c changes in patients treated with OW sema T2D, they did not include the 1.0 mg therapeutic dose for OW sema T2D and used a higher threshold for HbA1c control (9.0%) than is recommended by the ADA (7.0%).^23,24^ Describing the maintenance dose of 1.0 mg in real-world settings, using the HbA1c threshold of 7% is particularly important as it aligns with the different SUSTAIN clinical trials and label indications.^25^ Recognizing that there is a need to increase understanding of the effectiveness of OW sema T2D in the real world among patients who meet these criteria, the objective of this study is to describe patient characteristics and HbA1c changes for patients prescribed a 1.0 mg maintenance dose of OW sema T2D; providing a deeper understanding of the glycemic benefits of semaglutide and its role in the management of T2DM in the United States.

## METHODS

### Study Design

This was a retrospective study using medical claims, pharmacy claims, and enrollment information from January 1, 2017, to December 31, 2020 (study period). The data source for this study was the Optum Research Database, which contains de-identified enrollment, medical, and pharmacy claims data for commercial and Medicare Advantage patients. Institutional review board approval or waiver of approval was not required for this study because the study data were secondary and de-identified in accordance with the US Department of Health and Human Services Privacy Rule’s requirements for deidentification codified at 45 C.F.R. § 164.514(b). Throughout the study, patient privacy was preserved, and researchers complied strictly with all applicable Health Insurance Portability and Accountability Act data management rules and the 1964 Declaration of Helsinki and its later amendments or comparable ethical standards.

### Study Population

To be included in the study sample, patients had to have at least 1 claim for OW sema T2D between January 1, 2018, and December 31, 2019 (identification period). The date of the first claim was the index date. Patients had to be at least 18 years old, to be continuously enrolled in the health plan with medical and pharmacy benefits for at least 12 months before and including the index date (pre-index period) and at least 12 months following the index date (post-index period), and to have at least 1 claim indicating a diagnosis of T2DM during the 12 month pre-index period or the 12 month post-index date. Female patients with at least 1 code indicating pregnancy during the pre-index or post-index periods were excluded from the sample, as were patients with missing age, gender, or geographical region information. Patients were required to reach a 1.0 mg weekly maintenance dose, defined using days’ supply. The maintenance dose was identified as the designated dose on the pharmacy claim with the largest proportion of days covered starting on the index date. Patients with a 0.25 mg or 0.5 mg maintenance dose were excluded because these doses are prescribed in the same pen, and there is the potential for misclassification bias. A 2.0 mg dose had not been approved at the time of this study. Patients were excluded from the sample if there was no HbA1c value recorded during the pre-index period or if the pre-index HbA1c value was below 7%, as these patients do not meet the criteria for the study.

### Pre-index Demographic and Clinical Characteristics

Collected demographic data included age, gender, insurance type, and geographic region. The comorbidity score was calculated based on the presence of diagnosis codes on medical claims in the pre-index period. The Quan-Charlson comorbidity score was categorized into the following groups: 0, 1 to 2, 3 to 4, and 5 or greater.^26,27^ General comorbid conditions were defined using the Clinical Classifications Software managed by the Agency for Healthcare Research and Quality on the presence ICD-10-CM diagnosis codes in the pre-index period.

### Pre-index Medications

Medication classes were defined by the American Hospital Formulary Service.^28^ The proportion of patients with at least 1 pharmacy claim for hypotensive agents, antilipemic agents, renin-angiotensin-aldosterone system inhibitors, antibacterial agents, analgesics and antipyretics, devices, diabetic testing supplies, diuretics, sympatholytic agents, psychotherapeutic agents, and β-adrenergic agents during the pre-index period was calculated. The proportion of patients with at least one pharmacy claim for an antidiabetic agent, including metformin, SGLT-2 inhibitor, GLP-1 RA (excluding OW sema T2D), DPP-4 inhibitor, thiazolidinedione, sulfonylurea, and insulin during the pre-index period was also calculated.

### Outcomes

The index dose was identified by the dose on the pharmacy claim for the first OW sema T2D fill during the identification period. Provider specialty was identified using the specialty code on the index OW sema T2D pharmacy claim. The proportion of days covered was defined by the proportion of time over the course of a patient’s treatment that they were in possession of medication. It was calculated by dividing the number of days when OW sema T2D was available based on prescription fill dates and days’ supply by the number of days between the index date and the end of the post-index period.^29,30^ Overlapping days’ supply from early refills was accounted for and continued medication use was assumed during hospitalizations. The change in HbA1c was measured by the difference between the latest HbA1c value recorded during the post-index period and the last HbA1c value recorded during the pre-index period. For patients with more than 1 pre-index HbA1c lab measurement, the latest pre-index HbA1c value was used. Patients were considered persistent if there were at least 90 days of continuous treatment after the index date. For those whose HbA1c values were available, the change in HbA1c was calculated while the patient was still persistent with OW sema T2D.

### Analysis

Numbers and percentages were provided for categorical variables; means and SD were provided for continuous variables. Student’s *t* tests were used to determine whether the pre-index to post-index change in HbA1c differed from 0.

## RESULTS

### Patient Population

The final study sample included 2168 patients (**Supplementary Figure S1**). The mean (SD) age of the patient population was 58.2 years (11.0) (**Table 1**) with a median of 58.0 (not shown). Demographics showed that 52.2% of the population was male, 66.1% had commercial insurance, and 65.5% lived in the Southern US (**Table 1**). Most patients had at least 1 of the following comorbid conditions: lipid metabolism disorder (90.8%), hypertension (86.6%), diabetes with complications (86.8%), and other nutritional or endocrine or metabolic disorders (72.5%) (**Table 1**).

**Table 1.** Patient Demographics and Clinical Characteristics

	**Total (N = 2168)**
Age (y), mean (SD)	58.2 (11.0)
Age group (y), n (%)	
18-39	111 (5.1)
40-64	1389 (64.1)
65-74	541 (25.0)
≥75	127 (5.9)
Male gender, n (%)	1132 (52.2)
Insurance type, n (%)	
Commercial	1433 (66.1)
Medicare	735 (33.9)
Region, n (%)	
Northeast	200 (9.2)
Midwest	280 (12.9)
South	1420 (65.5)
West	268 (12.4)
Quan-Charlson Comorbidity Index score, mean (SD)	1.5 (1.6)
Lipid metabolism disorder	1968 (90.8)
Hypertension	1878 (86.6)
Diabetes mellitus with complications	1881 (86.8)
Other nutritional, endocrine, or metabolic disorder	1572 (72.5)
Valid n^a^	1772
Pre-index HbA1c,^b^ mean (SD)	8.8 (1.6)

^a^Patients with an HbA1c measurement in both the pre-index and post-index periods.

^b^Based on most recent HbA1c value measured during the pre-index period or on the index date.

Abbreviation: HbA1c, glycated hemoglobin.

### Pre-index Medications

**Figure 1.**
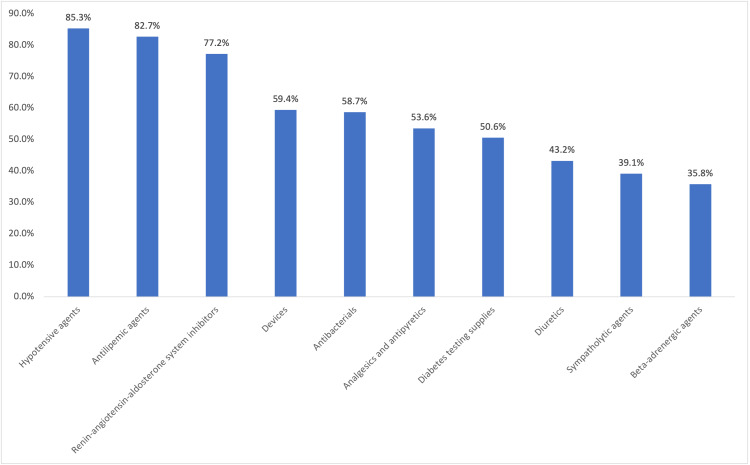
Top 10 Medication Classes Prescribed in the Pre-index Period (American Hospital Formulary Service)^a^ ^a^Including drugs filled on the index date.

**Figure 2.**
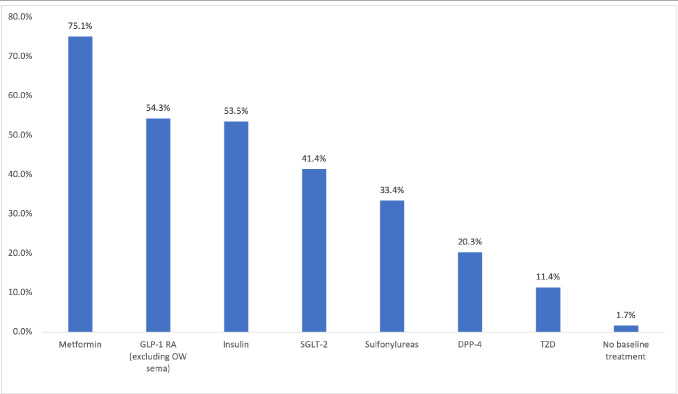
Diabetes Treatments Filled in the Pre-index Period Excluding OW Sema^a^ ^a^Including treatments filled on the index date. Abbreviations: DPP-4, dipeptidyl peptidase 4 inhibitors; GLP-1 RAs, glucagon-like peptide-1 receptor agonists; OW sema, once-weekly semaglutide for diabetes; SGLT-2, sodium-glucose cotransporter-2 inhibitors; TZD, thiazolidinedione.

During the pre-index period, patients were prescribed an average (SD) of 13.5 (6.2) different classes of medications. The most prescribed pre-index medication classes included hypotensive agents (85.3%), antilipemic agents (82.7%), and renin-angiotensin-aldosterone system inhibitors (77.2%) (**Figure 1**). The most prescribed pre-index diabetes medications include metformin (75.1%), GLP-1 RAs (excluding OW sema T2D, 54.3%), and insulin (53.5%) (**Figure 2**).

### Index Dose and Index Prescriber Specialty

Overall, 43.5% of patients were prescribed an initial weekly dose of 0.25/0.5 mg, whereas 56.5% of patients were prescribed an initial dose of 1.0 mg (**Table 2**). Overall, 33.9% of patients received their initial OW sema T2D prescription from an endocrinologist, 22.2% from a primary care provider, and 18.1% from a provider with an internal medicine specialty (**Table 2**).

**Table 2.** Index Dose, Prescriber Specialty, and Adherence

**Index OW sema Variable**	**Total (N = 2168)**
Index OW sema dose	
0.25/0.5 mg	942 (43.5)
1.0 mg	1226 (56.5)
Prescriber specialty^a^	
Primary care	481 (22.2)
Internal medicine	393 (18.1)
Endocrinologist	734 (33.9)
Other	428 (19.7)
Proportion of days covered, mean (SD)	0.75 (0.27)
Days covered with 1.0 mg maintenance dose, mean (SD)	236.1 (94.1)

^a^Patients with a neurologist, cardiologist, or unknown prescriber are not presented due to small sample size; the primary care category consists of clinic/family practice, family/general practice, OB/GYN, pediatrics, geriatrics; the other category includes specialties that are not part of any other category.

Abbreviation: OW sema, once-weekly semaglutide for diabetes.

### Proportion of Days Covered

The mean (SD) proportion of days covered with OW sema T2D was 0.75 (0.27). The mean (SD) days covered with a 1.0 mg maintenance dose was 236.1 (94.1) days (**Table 2**).

### HbA1c Changes

Among patients who had both a pre-index and post-index HbA1c (n = 1772), patients had a statistically significant mean (SD) HbA1c reduction of 1.2% (1.7) (*P* < .001) (**Table 3**). More than half (52.8%) of patients had a decrease in HbA1c of at least 1%. Among persistent patients (n = 1280), the mean (SD) reduction in HbA1c was 1.4% (1.6) (*P* < .001); 56.3% of persistent patients had a decrease in HbA1c of at least 1% (**Table 3**).

**Table 3.** Changes in Hemoglobin A1c

	**n (%)**	**Mean (SD) Change**
Patients with ≥1 HbA1c measurement in both pre-index and post-index periods	1772 (81.7)	–
Change in HbA1c between last pre-index and last post-index HbA1c measurement	1772^a^	−1.2 (1.7)*
Patients with HbA1c decrease ≥1.0%	935 (52.8)	–
Days from last pre-index to last post-index HbA1c measurement	–	318.0 (114.8)
Patients with ≥90 days continuous treatment with OW sema	1791 (82.6)	–
Change in HbA1c between last pre-index and last post-index HbA1c measurements^b^	1280^a^	−1.4 (1.6)*
Patients with HbA1c decrease ≥1.0%	721 (56.3)	–
Days from index date to last HbA1c measurement	–	257.1 (79.6)

^a^Valid n.

^b^Among persistent patients with >1 HbA1c measurement in both pre-and post-index periods

*P < .001.

Abbreviations: HbA1c, glycated hemoglobin; OW sema, once-weekly semaglutide for diabetes.

## DISCUSSION

This real-world study examined characteristics and HbA1c changes among patients with T2DM who initiated therapy with OW sema T2D at a maintenance dose of 1.0 mg weekly. The findings demonstrate a significant mean HbA1c reduction of 1.2%, showing a meaningful effect on glycemic control. The patient cohort was medically complex, with a mean of 13.5 (median, 13) different classes of medications and a range of comorbidities (ie, ≥70% had 1 comorbidity). This study demonstrates the effectiveness of a 1.0 mg maintenance dose of OW sema T2D in a real-world setting and among medically complex patients and gives an important context for prescribers using this medication with patients with T2DM.

The observed HbA1c reduction in this study (1.2%) is slightly lower than the reductions reported in the SUSTAIN clinical trials, where a 1.0 mg dose of OW sema reduced HbA1c by 1.5% to 1.8% after 30 to 56 weeks as shown by Meier in a meta-analysis of the SUSTAIN trials.^20^ This discrepancy may be attributed to the real-world setting of this study, where patient compliance and other uncontrolled variables can influence outcomes. Additionally, 82.6% of patients in this study were persistent with OW sema T2D for 90 days, whereas the clinical trial data were based on longer treatment durations. In addition, the SUSTAIN trials excluded patients with recent medication changes and comorbidities, such as heart failure and a history of pancreatitis, among others^11–16,31–33^; therefore, trial participants may have been less medically complex than the patients in the present study.

Despite the above-mentioned differences with clinical trials, our results are consistent with those presented in a meta-analysis of real-world studies that showed a reduction of 1.10% in HbA1c.^34^ However, this meta-analysis included studies from several countries, such as France, Italy, and Germany, and did not include any from the United States. Observing studies from the US reveals some differences. For example, Visaria et al conducted an evaluation of claims data among patients initiating OW sema and found an overall HbA1c reduction of 0.9%.^24^ The lower change in HbA1c in their study may be due to the inclusion of patients with controlled diabetes (pre-index HbA1c <7%), whereas our study focused on patients with higher HbA1c levels, providing greater opportunity for improvement. Similarly, a study by Frazer et al^23^ showed a reduction of 0.8% after 6 months of OW sema T2D; however, this study included a lower dose of OW sema T2D and allowed a different initial HbA1c threshold (<9%), which could explain the difference in results.

While this study provides relevant insights into the real-world use of OW sema T2D, some limitations should be acknowledged. First, claims data are collected for billing purposes and are not intended for research use, although this type of data has been broadly used to describe medication real-world usage. Second, the study population may not be representative of all T2DM patients. Third, selection bias is possible due to the exclusion of patients without available lab data. Fourth, medications were only identified from pharmacy claims and did not include outpatient claims. Fifth, data on social determinants of health and other information such as race/ethnicity, years living with T2DM, or socioeconomic profile are not available in claims data. Additionally, the analysis did not control for prior diabetes medications; patients could have initiated other medications along with OW sema T2D which may have contributed to the reduction in HbA1c. Finally, given the design of the study and the lack of a comparison arm, the findings are descriptive in nature and do not infer causal relationships.

## CONCLUSIONS

This study was conducted in a real-world setting with medically complex patients. Patients in this study experienced significant reductions in HbA1c following initiation of OW sema T2D. Persistent patients experienced greater reduction in HbA1c. Findings suggest that OW sema T2D at 1.0 mg maintenance dose is a valuable therapeutic option for glycemic control, reinforcing results from other real-world studies and clinical trials.

### Disclosures

This study was funded by Novo Nordisk, which had a role in the analysis and interpretation of the data. C.S., S.A., T.J.D., and J.N. are employees and shareholders of Novo Nordisk Inc. N.N.G., A.S., M.L., and E.B. are employees of Optum, which received funding from Novo Nordisk Inc. for conducting the study. M.S.F. was an employee of Optum at the time the study was conducted.

## Supplementary Material

Online Supplementary Material

## References

[1] Cho N. H., Shaw J. E., Karuranga S.. (2018). IDF Diabetes Atlas: global estimates of diabetes prevalence for 2017 and projections for 2045. Diabetes Res Clin Pract.

[2] Zheng Y., Ley S. H., Hu F. B. (2018). Global aetiology and epidemiology of type 2 diabetes mellitus and its complications. Nat Rev Endocrinol.

[3] American Diabetes Association (2024). Statistics about diabetes.

[4] Parker E. D., Lin J., Mahoney T.. (2024). Economic Costs of Diabetes in the U.S. in 2022. Diabetes Care.

[5] ElSayed N. A., Aleppo G.., American Diabetes Association Professional Practice Committee (2024). 6. Glycemic goals and hypoglycemia: Standards of Care in Diabetes—2024. Diabetes Care.

[6] ElSayed N. A., Aleppo G.., American Diabetes Association Professional Practice Committee (2024). 2. Diagnosis and classification of diabetes: Standards of Care in Diabetes—2024. Diabetes Care.

[7] ElSayed N. A., Aleppo G.., American Diabetes Association Professional Practice Committee (2024). Summary of revisions: Standards of Care in Diabetes—2024. Diabetes Care.

[8] ElSayed N. A., Aleppo G., Bannuru R. R., Bruemmer D., Collins B. S.., American Diabetes Association Professional Practice Committee (2024). 4. Comprehensive medical evaluation and assessment of comorbidities: Standards of Care in Diabetes—2024. Diabetes Care.

[9] ElSayed N. A., Aleppo G.., American Diabetes Association Professional Practice Committee (2024). 9. Pharmacologic approaches to glycemic treatment: Standards of Care in Diabetes—2024. Diabetes Care.

[10] Davies M. J., Aroda V. R., Collins B. S.. (2022). Management of hyperglycaemia in type 2 diabetes, 2022. A consensus report by the American Diabetes Association (ADA) and the European Association for the Study of Diabetes (EASD). Diabetologia.

[11] Ahmann A. J., Capehorn M., Charpentier G.. (2018). Efficacy and Safety of once-weekly semaglutide versus exenatide er in subjects with type 2 diabetes (SUSTAIN 3): a 56-week, open-label, randomized clinical trial. Diabetes Care.

[12] Ahrén B., Masmiquel L., Kumar H.. (2017). Efficacy and safety of once-weekly semaglutide versus once-daily sitagliptin as an add-on to metformin, thiazolidinediones, or both, in patients with type 2 diabetes (SUSTAIN 2): a 56-week, double-blind, phase 3a, randomised trial. Lancet Diabetes Endocrinol.

[13] Aroda V. R., Bain S. C., Cariou B.. (2017). Efficacy and safety of once-weekly semaglutide versus once-daily insulin glargine as add-on to metformin (with or without sulfonylureas) in insulin-naive patients with type 2 diabetes (SUSTAIN 4): a randomised, open-label, parallel-group, multicentre, multinational, phase 3a trial. Lancet Diabetes Endocrinol.

[14] Pratley R. E., Aroda V. R., Lingvay I.. (2018). Semaglutide versus dulaglutide once weekly in patients with type 2 diabetes (SUSTAIN 7): a randomised, open-label, phase 3b trial. Lancet Diabetes Endocrinol.

[15] Sorli C., Harashima S. ichi, Tsoukas G. M.. (2017). Efficacy and safety of once-weekly semaglutide monotherapy versus placebo in patients with type 2 diabetes (SUSTAIN 1): a double-blind, randomised, placebo-controlled, parallel-group, multinational, multicentre phase 3a trial. Lancet Diabetes Endocrinol.

[16] Zinman B., Bhosekar V., Busch R.. (2019). Semaglutide once weekly as add-on to SGLT-2 inhibitor therapy in type 2 diabetes (SUSTAIN 9): a randomised, placebo-controlled trial. Lancet Diabetes Endocrinol.

[17] Zhong P., Zeng H., Huang M., He G., Chen Z. (2021). Efficacy and safety of subcutaneous and oral semaglutide administration in patients with type 2 diabetes: a meta-analysis. Front Pharmacol.

[18] Zaazouee M. S., Hamdallah A., Helmy S. K.. (2022). Semaglutide for the treatment of type 2 diabetes mellitus: a systematic review and network meta-analysis of safety and efficacy outcomes. Diabetes Metab Syndr Clin Res Rev.

[19] Moiz A., Levett J. Y., Filion K. B., Peri K., Reynier P., Eisenberg M. J. (2024). Long-term efficacy and safety of once-weekly semaglutide for weight loss in patients without diabetes: a systematic review and meta-analysis of randomized controlled trials. Am J Cardiol.

[20] Meier J. J. (2021). Efficacy of semaglutide in a subcutaneous and an oral formulation. Front Endocrinol.

[21] Schneeweiss S., Patorno E. (2021). Conducting Real-world Evidence studies on the clinical outcomes of diabetes treatments. Endocr Rev.

[22] Sherman R. E., Anderson S. A., Dal Pan G. J.. (2016). Real-world evidence — what is it and what can it tell us?. N Engl J Med.

[23] Frazer M., Swift C., Sargent A.. (2023). Real-world HbA1c changes and prescription characteristics among type 2 diabetes mellitus patients initiating treatment with once weekly semaglutide for diabetes. J Diabetes Metab Disord.

[24] Visaria J., Uzoigwe C., Swift C., Dang-Tan T., Paprocki Y., Willey V. J. (2021). Real-world effectiveness of once-weekly semaglutide from a US commercially insured and Medicare Advantage population. Clin Ther.

[25] FDA Ozempic. Prescribing information.

[26] Quan H., Li B., Couris C. M.. (2011). Updating and validating the Charlson Comorbidity Index and score for risk adjustment in hospital discharge abstracts using data from 6 countries. Am J Epidemiol.

[27] Bayliss E. A., Ellis J. L., Shoup J. A., Zeng C., McQuillan D. B., Steiner J. F. (2012). Association of patient-centered outcomes with patient-reported and ICD-9-based morbidity measures. Ann Fam Med.

[28] Service A.H.F. AHFS Classification-Drug Assigmnents 2022.

[29] Peterson A. M., Nau D. P., Cramer J. A., Benner J., Gwadry-Sridhar F., Nichol M. (2007). A checklist for medication compliance and persistence studies using retrospective databases. Value Health.

[30] Ho P. M., Rumsfeld J. S., Masoudi F. A.. (2006). Effect of medication nonadherence on hospitalization and mortality among patients with diabetes mellitus. Arch Intern Med.

[31] Capehorn M. S., Catarig A. M., Furberg J. K., Janez A., Price H. C., Tadayon S.. (2020). Efficacy and safety of once-weekly semaglutide 1.0 mg vs once-daily liraglutide 1.2 mg as add-on to 1–3 oral antidiabetic drugs in subjects with type 2 diabetes (SUSTAIN 10). Diabetes Metab.

[32] Lingvay I., Catarig A. M., Frias J. P., Kumar H., Lausvig N. L., Le Roux C. W.. (2019). Efficacy and safety of once-weekly semaglutide versus daily canagliflozin as add-on to metformin in patients with type 2 diabetes (SUSTAIN 8): a double-blind, phase 3b, randomised controlled trial. Lancet Diabetes Endocrinol.

[33] Rodbard H. W., Lingvay I., Reed J.. (2018). Semaglutide added to basal insulin in type 2 diabetes (SUSTAIN 5): a randomized, controlled trial. J Clin Endocrinol Metab.

[34] Wang L., Yang Y., Yu J.. (2022). Efficacy and safety of anti-PD -1/ PD-L1 in combination with chemotherapy or not as first-line treatment for advanced non-small cell lung cancer: a systematic review and network meta-analysis. Thorac Cancer.

