# Supplementary material for: Real-World HbA1c Changes Among Type 2 Diabetes Mellitus Patients Initiating Treatment With a 1.0 Mg Weekly Dose of Semaglutide for Diabetes
Source: J Health Econ Outcomes Res. 2024 Nov 4;11(2):118–24. doi: 10.36469/001c.124111 (PMC11539928; doi:10.36469/001c.124111)
Supplement: Online Supplementary Material [file jheor_2024_11_2_124111_251989.pdf]

Journal of Health Economics and Outcomes Research

Online Supplementary Material

Real-World HbA1c Changes Among Type 2 Diabetes Mellitus Patients Initiating Treatment With a 1.0 mg Weekly Dose of Once-Weekly Semaglutide for Diabetes. *JHEOR*. 2024;11(2):118-124. doi:10.36469/jheor.2024.124111

Table S1: Patient Sample Selection

This supplementary material has been provided by the authors to give readers additional information about their work.

This is an open-access article distributed under the terms of the Creative Commons Attribution 4.0 International License (CCBY-4.0). View this license's legal deed at http://creativecommons.org/licenses/by/4.0 and legal code at http://creativecommons.org/licenses/by/4.0/legalcode for more information.

Figure 1. Patient Sample Selection

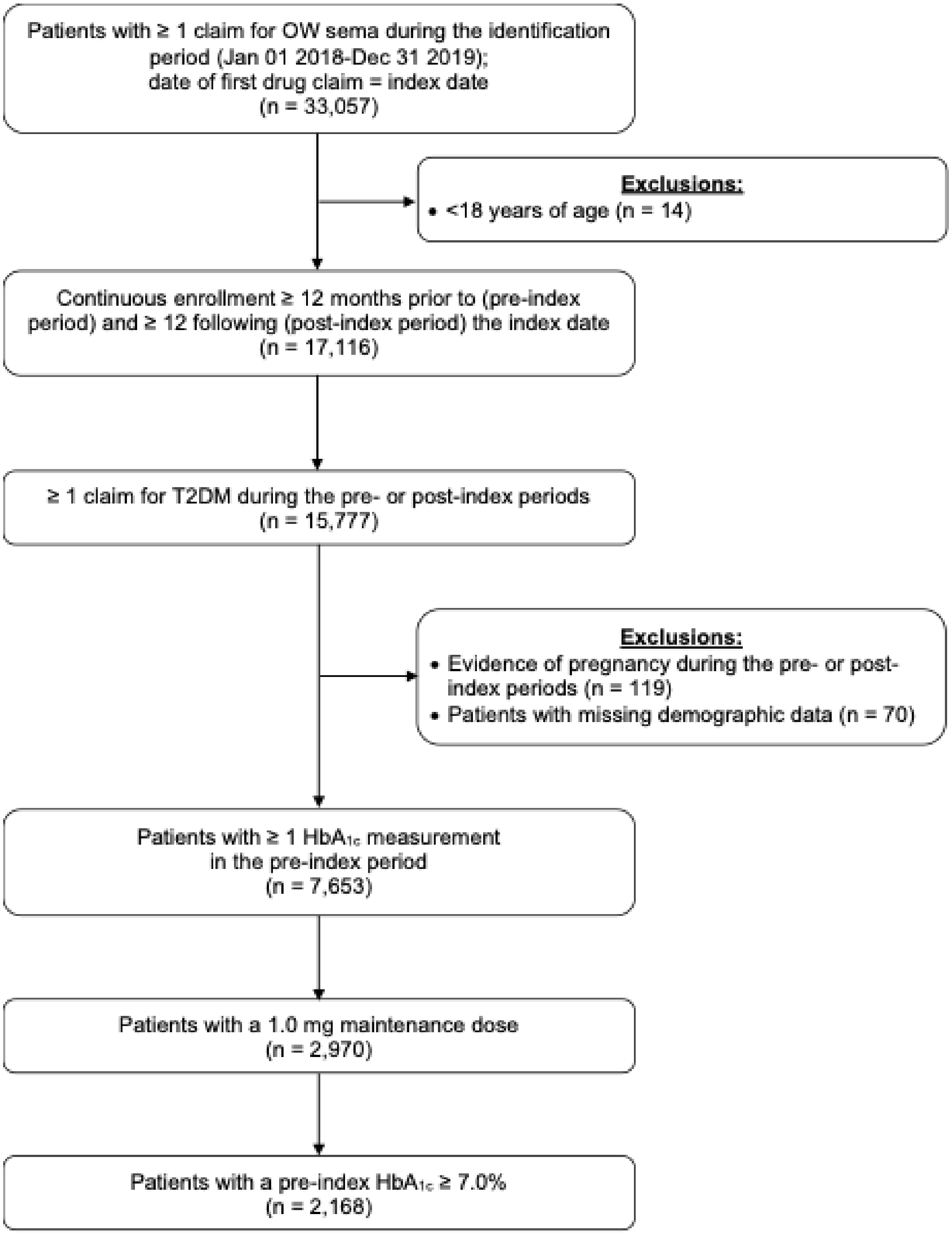

Abbreviations: HbA1c, glycated hemoglobin; OW sema, once-weekly semaglutide for diabetes; T2DM, type 2 diabetes mellitus.
